# The Roles of Liver Inflammation and the Insulin Signaling Pathway in PM2.5 Instillation-Induced Insulin Resistance in Wistar Rats

**DOI:** 10.1155/2021/2821673

**Published:** 2021-10-29

**Authors:** Zhihua Zhang, Shujun Hu, Ping Fan, Ling Li, Shanshan Feng, Huabing Xiao, Lingyan Zhu

**Affiliations:** ^1^Queen Mary School, Nanchang University, Nanchang 330006, China; ^2^Department of Endocrinology, The First Affiliated Hospital of Nanchang University, Nanchang 330006, China; ^3^Department of Endocrinology and Metabolism, Tongji Hospital, School of Medicine, Tongji University, Shanghai 200065, China; ^4^The People's Hospital of Yudu County, Yudu 342300, China

## Abstract

To elucidate the mechanism of how the liver participates in PM2.5-caused insulin resistance. A novel Wistar rat model was developed in this study by instilling a suspension of lyophilized PM2.5 sample (2.5 mg/kg, 5 mg/kg, or 10 mg/kg) collected from the atmosphere. Systemic insulin resistance indicators, including serum fasting blood glucose (FBG), fasting insulin (FINS), Homeostatic Model Assessment for Insulin Resistance (HOMA-IR), and hemoglobin A1 (HbA1), were upregulated by the PM2.5 instillation. The area under the curve (AUC_glu_) calculated by intraperitoneal glucose tolerance testing (IPGTT) was also significantly greater in the PM2.5 instillation groups. Additionally, PM2.5 instillation was found to cause liver damage and inflammation. The serum levels of aspartate aminotransferase (AST), alanine aminotransferase (ALT), total bilirubin (TBIL), tumor necrosis factor-*α* (TNF-*α*), and interleukin-6 (IL-6) were significantly elevated by PM2.5 instillation. PM2.5 also triggered IL-6 and TNF-*α* transcription but inhibited mRNA synthesis and suppressed signaling activation of the insulin-phosphoinositide 3-kinase- (PI3K-) Akt-glucose transporter 2 (GLUT2) pathway in the rat liver by reducing the ratio of phosphorylated Akt to phosphorylated insulin receptor substrate 1 (IRS-1). Thus, PM2.5-induced inflammation activation and insulin signaling inhibition in the rat liver contribute to the development of systemic insulin resistance.

## 1. Introduction

According to the World Health Organization, the global prevalence of diabetes in adults over 18 years old increased from 4.7% in 1980 to 8.5% in 2014, while the prevalence of diabetes in China had already reached 9.7% in 2010 [1]. Diabetes can lead to blindness, renal failure, cardiovascular disease, and reduced limb amputation; therefore, the burden caused by diabetes to families is tremendous. Large numbers of related studies have shown that the main mechanism involved in the development and progression of type 2 diabetes is insulin resistance, which implies a significant reduction in the physiological effects of uptake and utilization of glucose [2]. Thus, there is an urgent need to better understand the underlying mechanism in order to design efficient insulin resistance prevention strategies. On the other hand, with the development of modern industries and urbanization, air pollution has become an issue throughout the world [3]. Meanwhile, more and more studies have demonstrated the adverse effects of ambient air pollutants such as particulate matter with a diameter less than 2.5 *μ*m (PM2.5) on human health. A strong association between PM2.5 exposure and diabetes prevalence has been established, suggesting that ambient air pollution may contribute to the increased prevalence of diabetes in the adult U.S. and Asian populations [4, 5]. Moreover, animal experimental studies have shown that PM2.5 exposure leads to systemic inflammation and insulin resistance [6–8], but the roles of target organs besides the pancreas, including the liver, adipose tissue, and skeletal muscle, on PM2.5-induced insulin resistance still remain controversial. The liver is one of the most important organs involved in insulin resistance, and some studies have shown that PM2.5 exposure induces liver damage [8, 9]; however, another study has found no significant effect of PM2.5 on the liver [10]. To systematically analyze the hepatic mechanism involved in the development of systemic insulin resistance, we performed PM2.5 instillation (0, 0.75, 2.5, 5, or 10 mg/kg) in Wistar rats for 8 weeks. Systemic insulin resistance indicators, including serum fasting blood glucose (FBG), fasting insulin (FINS), Homeostatic Model Assessment for Insulin Resistance (HOMA-IR), and hemoglobin A1 (HbA1), were examined. The area under the curve (AUC_glu_) calculated by intraperitoneal glucose tolerance testing (IPGTT) in the different PM2.5 instillation groups was also measured. In addition, the serum levels of aspartate aminotransferase (AST), alanine aminotransferase (ALT), total bilirubin (TBIL), tumor necrosis factor-*α* (TNF-*α*), and interleukin-6 (IL-6) as well as the hepatic mRNA expression of IL-6 and TNF-*α* were determined. Furthermore, the impact of PM2.5 exposure on the synthesis of insulin-phosphoinositide 3-kinase (PI3K-) Akt-glucose transporter 2 (GLUT2) signaling pathway molecules in the rat liver at the mRNA level was analyzed. Finally, the PM2.5-induced phosphorylation of Akt and insulin receptor substrate 1 (IRS-1) in the rat liver was evaluated and compared by western blotting.

## 2. Materials and Methods

### 2.1. PM2.5 Collection and Suspension Preparation

The PM2.5 samples were collected from December 2016 to March 2017 at Xinxiang Medical College, which is located close to the center of Xinxiang City (Xinxiang, Henan, China). The sampling site is surrounded by residential areas and is far away from major roads and factories. After sampling, the filter of the PM2.5 high-volume air sampler (Tisch Environmental, USA) was cut off and sonicated in sterile double-distilled water using a UC-6B ultrasonicator (Shanghai, China) for 3 × 15 min. The PM2.5 eluted from the filter was then lyophilized, weighed, and stored at −20°C until further use. Scanning electron microscopy was also conducted to confirm that the particulate matter collected had a diameter of no more than 2.5 *μ*m.

### 2.2. Animal Models

Sixty healthy Wistar rats, weighing 180–220 g, were purchased from Charles River, China. To avoid sex-dependent differences, only male mice were included in the study. All experiments described in the experimental protocol were approved by the Institutional Review Committee for Animal Care and Use at Xinxiang Medical College (Xinxiang, Henan, China), and all experimental procedures were performed to minimize animal suffering. The health and behavior of the animals were checked daily. The rats were housed together and acclimatized in a freestanding clean room for 1 week (22–28°C, 60% humidity, *ad libitum* access to filtered water and food, 12 h light/12 h dark cycle). Sixty rats were then randomly allocated into five groups (12 rats/group), including control, 0.75 mg/kg, 2.5 mg/kg, 5 mg/kg, and 10 mg/kg PM2.5 instillation groups. Before exposure, the PM2.5 lyophilized powder taken from a refrigerator was weighed, and the corresponding concentration of PM2.5 was resuspended in saline. To avoid aggregation, particle suspensions were always sonicated for 3 × 15 min and vortexed. By using noninvasive inhalation tracheal instillation, the rats in each dosage group were infused with PM2.5 three times a week for 8 weeks [10]. The rats in the control group were given the same volume of normal saline via tracheal instillation. No rats died during this experiment.

### 2.3. IPGTT and Sample Collection

After PM2.5 instillation, IPGTT of rats was performed in each group, and the AUC_glu_ was calculated [11]. Venous blood from rats that had fasted for 4.5 h was obtained from a small tail clip, and the fasting glucose level (0 min) measurements were conducted with an ACCU-CHEK glucometer (Roche, Shanghai, China). After 50% glucose solution (2 g/kg body weight) was injected intraperitoneally, the blood glucose values were measured at 5, 10, 30, 60, and 120 min postadministration. The AUC_glu_ was calculated using the following formula: AUC_glu_ = (0 min + 5 min)/24 + (5 min + 10 min)/24 + (10 min + 30 min)/6 + (30 min + 60 min)/4 + (60 min + 120 min)/2. After IPGTT, the animals were anesthetized with ether and sacrificed by femoral artery terminal exsanguination. The blood was collected for serum isolation and stored at −70°C until use. At the same time, the liver was removed and spliced into two equal parts. After weighing, one part was fixed in 4% buffered paraformaldehyde, and the other part was frozen at −70°C.

### 2.4. Hematoxylin and Eosin (HE) Staining

Briefly, rat liver samples fixed in 4% buffered paraformaldehyde were dehydrated in a series of ethanol solutions, cleared in xylene, and embedded in paraffin. The paraffin-embedded tissues were sectioned into 4 *μ*m thick slices using a rotary microtome (Leica RM 2016, Wetzlar, Germany) and subjected to HE staining with hematoxylin for 5–7 min at room temperature. After rinsing, the slices were stained with eosin for 2 min at room temperature. Micrographs under 200x magnification were randomly selected and captured using a light microscope.

### 2.5. Circulating Inflammation-Related and Biochemical Biomarker Measurements

The FBG, ALT, AST, and TBIL levels were determined by Chemoray (Rayto, Shenzhen, China). The FINS, IL-6, and TNG-*α* levels were measured by enzyme-linked immunosorbent assay (ELISA) kits, according to the manufacturer's instructions (Elabscience Biotechnology, Wuhan, China). The HbA1c level was determined by ELISA, according to the manufacturer's instructions (USCN Business, Wuhan, China). The HOMA-IR was calculated by the following formula: HOMA‐IR = FBG × FINS/22.5 [12].

### 2.6. Quantitative Polymerase Chain Reaction (qPCR)

Total RNA was isolated from whole liver lysates by TRIzol, according to the manufacturer's instructions (TRIzol, Aidlab, Beijing). RNA was reverse-transcribed using the reverse transcriptase M-MLV (RNase H) (FulenGen, Guangzhou, China). The synthesized cDNA was subjected to qPCR using 2x All-in-One qPCR Mix (Vazyme, Nanjing, China) in triplicate, according to the manufacturer's instructions. The *β*-actin gene was used as an internal control to normalize the expression of target genes, and the specific primers are listed in Table 1. The melting curves and the *E* = 2^−△△*C*^*t* algorithm were analyzed by LightCycler software (Roche Diagnostics).

### 2.7. Western Blot Analysis

Total protein of rat liver tissue in each group was extracted using radioimmunoprecipitation assay lysis buffer. The protein concentration was determined using a bicinchoninic acid protein concentration quantification kit (Beyotime Biotechnology, Shanghai, China). Denatured proteins were separated by sodium dodecyl sulfate-polyacrylamide gel electrophoresis and transferred to a polyvinylidene difluoride membrane. The membranes were blocked with 5% nonfat dry milk in tris-buffered saline containing 0.05% Tween 20 or 1–3% bovine serum albumin and probed with primary antibodies overnight at 4°C. The primary antibodies used included IRS-1, Akt (1 : 1000 dilution, Proteintech Inc., Wuhan, China), p-IRS-1 (1 : 1000 dilution, Cell Signaling, USA), p-Akt^Ser473^ (1 : 2000 dilution, Abcam, USA), and *β*-actin (1 : 200 dilution, Boster Biological Technology Co. Ltd., Wuhan, China). Following washing, the membranes were incubated with goat horseradish peroxidase-conjugated secondary antibodies (1 : 50,000 dilution, Boster Biological Technology Co. Ltd., Wuhan, China). The reactions were detected by using an Enhanced Chemiluminescence Western Blot detection kit (Thermo Fisher, USA), and the detected bands were visualized via exposure to an X-ray beam in a dark room.

### 2.8. Statistical Analysis

Data were analyzed by SPSS20.0 (IBM Corp., Armonk, NY, USA). For the normally distributed measurement data, the results were presented as the mean ± standard error, and the Levene test was used for the homogeneity of variance. One-way analysis of variance was used for multiple group comparisons, and the least significant difference test was used for pairwise comparisons between groups. Nonparametric tests were used for measurement data that did not meet the assumptions of equal variance. *p* < 0.05 was set to indicate a significant difference.

## 3. Results

### 3.1. PM2.5 Instillation-Induced Insulin Resistance in Rats

**Figure 1:**
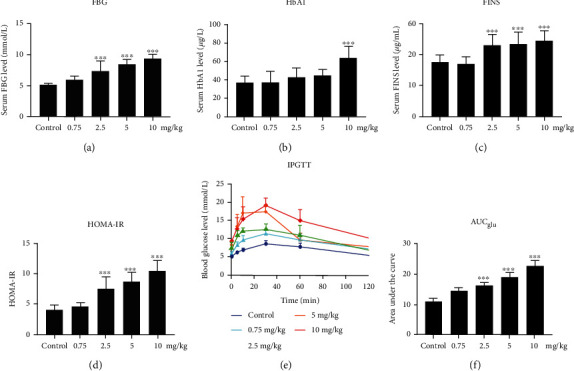
Effect of PM2.5 exposure on systemic insulin resistance indicators in Wistar rats. Experiments were performed after PM2.5 instillation at different dosages or saline instillation (control) for 8 weeks. Values are shown as the mean ± standard error of 12 rats/group. ^∗^*p* < 0.05, and ^∗∗^*p* < 0.01. (a) Fasting blood glucose (FBG), (b) fasting insulin (FINS), (c) hemoglobin A1c (HbA1c), (d) Homeostatic Model Assessment for Insulin Resistance (HOMA-IR), and (e) intraperitoneal glucose tolerance testing (IPGTT) curves. (f) The area under the curve (AUC_glu_).

To study the effect of PM2.5 instillation on systemic insulin resistance in rats, the levels of FBG, FINS, HbA1c, and HOMA-IR were examined in all five groups (Figures 1(a)–1(d) and Table 2(a)). Compared to the control group, the levels of FBG, FINS, and HOMA-IR started to be significantly higher in the 2.5 mg/kg PM2.5 group and continued to the 10 mg/kg PM2.5 group (*p* < 0.05). Compared to the 0.75 mg/kg PM2.5 group, the FBG, FINS, and HOMA-IR levels in the 2.5, 5, and 10 mg/kg PM2.5 groups were all significantly elevated (*p* < 0.05). The FINS and HOMA-IR levels in the 5 mg/kg PM2.5 group were significantly higher (*p* < 0.05) than those in the 2.5 mg/kg PM2.5 group. The FBG and HOMA-IR but not the FINS level in the 10 mg/kg PM2.5 group were significantly elevated compared to those in the 2.5 mg/kg PM2.5 group. There were no significant differences in the FBG, FINS, and HOMA-IR levels between the 5 and 10 mg/kg PM2.5 groups. Only the HbA1c level in the 10 mg/kg PM2.5 group was significantly increased when compared to each of the other four groups (*p* < 0.05). Next, we performed IPGTT and calculated the AUC_glu_ for the rats in each group administered with different dosages of PM2.5 instillation (Figures 1(e) and 1(f) and Table 2(b)). Compared to the control group, the glucose levels in the 2.5, 5, and 10 mg/kg PM2.5 groups at each time point were all significantly elevated (*p* < 0.05). Similarly, the AUC_glu_ in the 2.5, 5, and 10 PM2.5 groups were also significantly higher than that of the control group (*p* < 0.05). Thus, PM2.5 instillation in rats induced insulin resistance in a dose-dependent manner.

### 3.2. PM2.5 Instillation Induced Liver Injury and Inflammation in Rats

**Figure 2:**
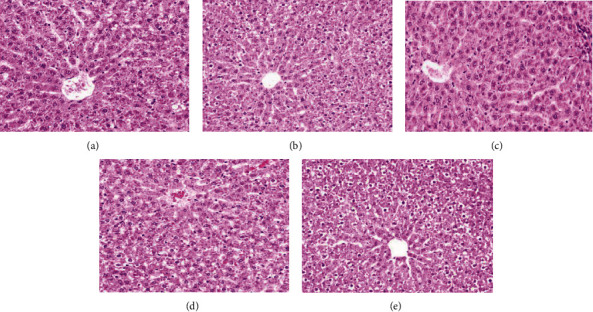
Effect of PM2.5 exposure on liver damage. (a–e) Representative images of liver hematoxylin and eosin staining in the control group (a), 0.75 mg/kg PM2.5 group (b), 2.5 mg/kg PM2.5 group (c), 5 mg/kg PM2.5 group (d), and 10 mg/kg PM2.5 group (e). Original magnification, 200x.

**Figure 3:**
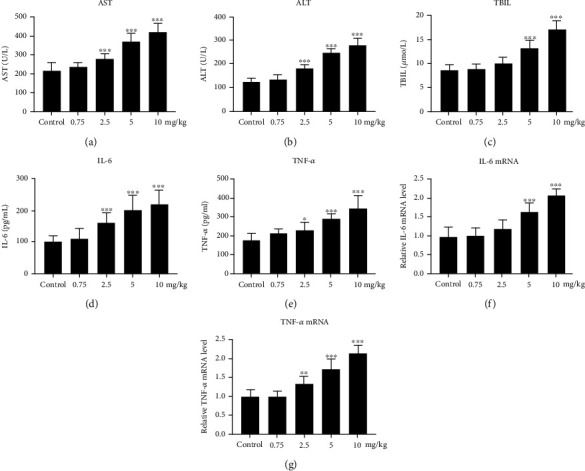
Effect of PM2.5 exposure on the expression of liver injury and inflammation-related biomarkers. (a–e) The circulating concentrations of AST (a), ALT (b), TBIL (c), IL-6 (d), and TNF-*α* (e) in response to PM2.5 exposure. (f, g) PM2.5-induced cytokine mRNA expression of IL-6 (f) and TNF-*α* (g) in the rat liver. Values are shown as the mean ± standard error of 12 rats/group. ^∗^*p* < 0.05, and ^∗∗^*p* < 0.01.

At first, we found that PM2.5 instillation induced liver damage in a dose-dependent manner according to the pathological analysis with HE staining of the liver sections (Figures 2(a)–2(e)). Compared to the control group, as the dose of PM2.5 instillation increased, the amounts of inflammatory cell infiltration and ballooning of hepatocytes increased. Next, we examined the serum level changes of the liver injury and inflammation-related biomarkers, including AST, ALT, TBIL, IL-6, and TNF-*α* (Figures 3(a)–3(e) and Table 3(a)). Compared to the control group, the ALT, AST, IL-6, and TNF-*α* serum levels were significantly elevated (*p* < 0.05), starting from 2.5 mg/kg PM2.5 instillation. The serum level of TBIL started to be significantly increased with 5 mg/kg PM2.5 instillation (*p* < 0.05). Compared to the 0.75 mg/kg PM2.5 group, the ALT and IL-6 serum levels started to be significantly higher with 2.5 mg/kg PM2.5 instillation (*p* < 0.05), while the serum levels of AST, TBIL, and TNF-*α* started to be significantly elevated with 5 mg/kg PM2.5 instillation (*p* < 0.05). Compared to the 2.5 mg/kg group, the ALT, AST, TBIL, and TNF-*α* levels in the 5 and 10 mg/kg PM2.5 groups were all significantly higher (*p* < 0.05), whereas the serum level of IL-6 was only significantly increased in the 10 mg/kg PM2.5 group (*p* < 0.05).

To confirm the protein level changes of the proinflammatory cytokines in PM2.5-instilled rats, we further examined the mRNA expression of IL-6 and TNF-*α* in the rat liver of each group by qPCR (Figures 3(f) and 3(g) and Table 3(b)). Compared to the control and 0.75 mg/kg PM2.5 groups, IL-6 mRNA expression was significantly increased in the 5 and 10 mg/kg PM2.5 groups (*p* < 0.05); TNF-*α* mRNA expression was significantly higher in the 2.5, 5, and 10 mg/kg PM2.5 groups (*p* < 0.05). Compared to the 2.5 mg/kg group, IL-6 and TNF-*α* mRNA expressions were both significantly elevated in the 5 and 10 mg/kg PM2.5 groups (*p* < 0.05). There was also an elevation in the IL-6 and TNF-*α* mRNA expression in the 10 mg/kg PM2.5 group, compared to the 5 mg/kg PM2.5 group (*p* < 0.05), indicating that PM2.5 induced proinflammatory cytokine transcription in the rat liver.

### 3.3. PM2.5 Instillation Inhibited the Synthesis of Hepatic Insulin Signaling Pathway Molecules

**Figure 4:**
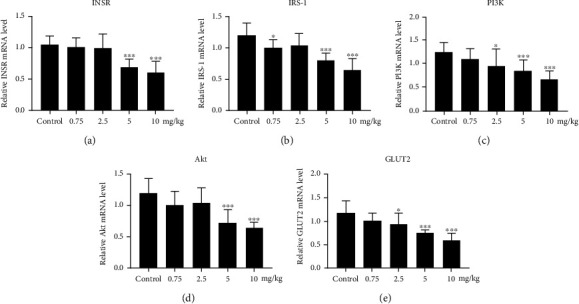
Effect of PM2.5 exposure on the expression of insulin-PI3K-Akt-GLUT2 signaling pathway molecules. (a–e) PM2.5-induced mRNA expression changes of INSR (a), IRS-1 (b), PI3K (c), Akt (d), and GLUT2 (e). Values are shown as the mean ± standard error of 12 rats/group. ^∗^*p* < 0.05, and ^∗∗^*p* < 0.01.

Inhibition of the insulin-PI3K-Akt-GLUT2 signaling pathway has been reported to be associated with insulin resistance [13–16]. To further investigate the mechanism underlying PM2.5 instillation-induced insulin resistance in the rat liver, the mRNA expression of the insulin-PI3K-Akt-GLUT2 signaling pathway molecules was examined by qPCR (Figures 4(a)–4(e) and Table 4). Compared to the control group, the GLUT2 mRNA level was significantly lower in the 2.5, 5, and 10 mg/kg PM2.5 groups (*p* < 0.05), while the insulin receptor (INSR), IRS-1, PI3K, and Akt mRNA levels were significantly lower in the 5 and 10 mg/kg PM2.5 groups (*p* < 0.05). Compared to the 0.75 mg/kg PM2.5 group, the INSR, IRS-1, Akt, and GLUT2 mRNA levels in the 5 and 10 mg/kg PM2.5 groups were significantly decreased (*p* < 0.05), while the PI3K mRNA level was significantly decreased in the 10 mg/kg PM2.5 group (*p* < 0.05). Compared to the 2.5 mg/kg PM2.5 group, the INSR, IRS-1, and Akt mRNA expression levels in the 5 and 10 mg/kg PM2.5 groups were significantly lower, while the PI3K and GLUT2 mRNA levels were significantly lower in the 10 mg/kg PM2.5 group (*p* < 0.05). Compared to the 5 mg/kg PM2.5 group, there was no significant difference in the INSR, IRS-1, PI3K, Akt, or GLUT2 mRNA level in the 10 mg/kg PM2.5 group. Decreased expression of these molecules at the mRNA level revealed that PM2.5 instillation may induce insulin resistance by inhibiting the transcription of insulin-PI3K-Akt-GLUT2 signaling pathway molecules.

### 3.4. PM2.5 Instillation Induced Phosphorylation of Insulin Signaling Pathway Proteins

**Figure 5:**
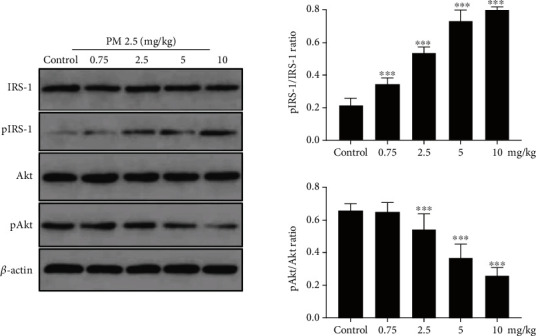
Effect of PM2.5 exposure on the phosphorylated and total protein expression changes in the rat liver. (a–d) Representative western blotting bands of IRS-1 (a), p-IRS-1 (b), Akt (c), and p-Akt (d) proteins. (e) Statistical analysis. Values are shown as the mean ± standard error of 12 rats/group. ^∗^*p* < 0.05, and ^∗∗^*p* < 0.01.

Phosphorylation of key serine residues is another indicator of insulin-PI3K-Akt-GLUT2 signaling pathway function. For example, serine phosphorylation of Akt is accompanied by insulin receptor activation. In contrast, serine phosphorylation of IRS proteins can decrease their capacity to attract downstream PI3K, resulting in signaling inhibition [13–16]. Thus, we next examined the changes in IRS-1, p-IRS-1, Akt, and p-Akt protein expression by western blot analysis (Figures 5(a)–5(d)). The ratio of p-IRS-1/ISR-1 increased, while the ratio of p-Akt/Akt decreased as the PM2.5 instillation dose increased (Figure 5(e) and Table 5). Compared to the control group, the p-IRS-1/ISR-1 ratios were significantly higher in all study groups (*p* < 0.05), while the p-Akt/Akt ratio started to be significantly decreased in the 2.5 mg/kg PM2.5 group (*p* < 0.05). Compared to the 0.75 mg/kg PM2.5 group, the ratios of p-IRS-1/ISR-1 and p-Akt/Akt in the 2.5, 5, and 10 mg/kg PM2.5 groups were all significantly different (*p* < 0.05). Compared to the 2.5 mg/kg PM2.5 group, the ratios of p-IRS-1/ISR-1 and p-Akt/Akt in the 5 and 10 mg/kg PM2.5 groups were all significantly different (*p* < 0.05). There was also a significant difference in the ratios of both p-IRS-1/ISR-1 and p-Akt/Akt between the 5 and 10 mg/kg PM2.5 groups (*p* < 0.05). Collectively, our results indicate that the phosphorylation of insulin signaling pathway proteins induced by PM2.5 instillation contributed to insulin resistance in the rat liver.

## 4. Discussion

Increasing numbers of experimental animal research studies have demonstrated the impact of PM2.5 inhalation on the development of insulin resistance and diabetes [17]. For example, long-term exposure to PM2.5 in C57BL/6 mice resulted in impaired glucose tolerance and resistance to insulin [8]. In a Sprague-Dawley rat model, exposure to PM2.5 significantly activated the oxidative response and inflammation in the pancreas, leading to reduced levels of GLUT2 [18]. In the present study, for the first time, PM2.5-induced insulin resistance was established in a Wistar rat model. In animal models, intratracheal inhalation and instillation are the main techniques of PM2.5 exposure [17]. Inhalation exposure is a physiological method that requires both an expensive exposure chamber and technical expertise. In contrast, intratracheal instillation includes direct application of the material to the trachea. It enables more control over the concentration and location of the material, and it is cheaper. The disadvantages of intratracheal instillation include its invasive and nonphysiological features. To overcome these drawbacks, in this study, a noninvasive intratracheal instillation method was used. This method caused less stress to the animals as it is not invasive; therefore, it is more reliable and the effectiveness was proved by our consistent results. The PM2.5-induced systemic insulin resistance was initially observed after exposure for 8 weeks. Systemic insulin resistance indicators, including FBG, FINS, HOMA-IR, and HbA1, were upregulated as the PM2.5 instillation concentration was increased. At the same time, the AUC_glu_ calculated by IPGTT with different dosages of PM2.5 instillation was also significantly greater than that of the control group.

The liver is one of the most important organs involved in insulin resistance, and the inhibition or defects of the insulin-PI3K-Akt signaling pathway in the liver may be the underlying mechanism [13–16]. Once activated by an insulin signal, the main downstream effector Akt enters into the cytoplasm, where glycogen synthase kinase 3 (GSK3) is phosphorylated and inactivated, which in turn promotes glycogen synthesis. In addition, inactivation of GSK3 by Akt results in the dephosphorylation of eukaryotic translation initiation factor 2 subunit beta and the storage of amino acids. PI3K and Akt are also known to play a role in insulin-induced glucose uptake into cells by translocation of the glucose transporter GLUT2. In line with these findings, in our study, the hepatic insulin-PI3K-Akt-GLUT2 signaling pathway was found to be consistently inactivated at both the mRNA expression and protein phosphorylation levels by PM2.5.

It has become increasingly evident that insulin resistance is often associated with the proinflammatory cytokine response in insulin-sensitive tissues including the liver, which may lead to a decreased insulin sensitivity [19–21]. For instance, TNF-*α* stimulation leads to serine phosphorylation of IRS-1, which attenuates its ability to transduce insulin-mediated cellular events [22]. Furthermore, mice genetically deficient in TNF-*α* or the TNF receptor 1 gene do not develop insulin resistance, even under elevated fatty or obese circumstances [23]. Treatment of cultured 3T3-L1 adipocytes with TNF-*α* also led to reduced expression of the INSR, IRS-1, and GLUT2 genes as well as decreased insulin-stimulated glucose uptake [24]. Here, we found that after 8 weeks of PM2.5 instillation, the rat liver developed inflammation, as shown by the HE staining experiments. The extent of the injury and inflammation increased as the dose of the PM2.5 instillation increased. The liver damage markers ALT, AST, and TIBL also increased, which further indicated liver injury. In addition to liver injury, there was also systemic as well as liver inflammation, with an increase of IL-6 and TNF-*α* at both the protein and mRNA levels. These observations are consistent with some previous studies, in which IL-6 has been reported to be elevated after PM2.5 exposure [7]. Future studies are warranted to determine the acute effect of PM2.5 on the interaction between inflammation and the insulin signaling pathway. At the same time, other insulin-sensitive tissues, such as adipose tissue and skeletal muscle, should also be further evaluated using the same model.

However, our study had several limitations. For example, due to the constraints of their availability, more advanced methods, such as a versatile aerosol concentration enrichment system, were not used. Furthermore, we did not perform *in vitro* cell biology experiments to observe and study the effect of PM2.5 on the insulin signaling pathway in human liver cells to determine if the results from our rat study are consistent or to clarify its potential clinical significance. Nevertheless, to the best of our knowledge, this is the first systematic study investigating the impact of PM2.5 on insulin resistance-related hepatic inflammation and the insulin signaling pathway in a rat model. Our findings also suggest a link between air pollution and hepatic metabolic abnormalities, which is important information for public health agencies to assess risk to humans.

## Figures and Tables

**Table 1:** PCR primers.

Primers	Sequences	Product size
*β*-Actin-F *β*-Actin-R	5′-CACGATGGAGGGGCCGGACTCATC-3′	240 bp
	5′-TAAAGACCTCTATGCCAACACAGT-3′	

AKT1-F AKT1-R	5′-GCTCTTCTTCCACCTGTCTCG-3′	186 bp
	5′-CACAGCCCGAAGTCCGTTA-3′	

PI3K-F PI3K-R	5′-GACAGGCACAACGACAAC-3′	214 bp
	5′-AAGCCCTAACGCAGACAT-3′	

INSR-F INSR-R	5′-AATGAGGAATGTGGGGACGT-3′	202 bp
	5′-GTTCTGAACAGTTGCCCAGG-3′	

IRS1-F IRS1-R	5′-GTCCAGACTCCTCCAACCTC-3′	239 bp
	5′-AGACCCACCTCCAATGTCAG-3′	

GLUT2-F GLUT2-R	5′-AGTCACACCAGCACATACGA-3′	170 bp
	5′-TGGCTTTGATCCTTCCGAGT-3′	

IL-6-F IL-6-R	5′-GTTGCCTTCTTGGGACTGATG-3′	102 bp
	5′-TACTGGTCTGTTGTGGGTGGT-3′	

TNF-*α*-F TNF-*α*-R	5′-GAAACAGTCTGCGAGGTGTG-3′	158 bp
	5′-TTCTTCTTGCAGCCACACAC-3′	

**Table:** (a) Serum concentrations of FBG, HbA1c, INS, and HOMA-IR in rats ($\overset{®}{x}\pm s$)

	FBG (mmol/L)	HbA1 (*μ*g/mL)	INS (ng/mL)	HOMA-IR
Control group	5.20 ± 0.29	37.02 ± 7.35	17.74 ± 2.38	4.119 ± 0.748
0.75 mg/kg PM2.5 group	6.02 ± 0.48	37.93 ± 11.32	17.39 ± 2.13	4.651 ± 0.623
2.5 mg/kg PM2.5 group	7.46 ± 1.48^ab^	43.05 ± 9.51	23.04 ± 3.50^ab^	7.632 ± 1.860^ab^
5 mg/kg PM2.5 group	8.54 ± 0.68^abc^	44.86 ± 6.67	23.61 ± 3.63^ab^	8.925 ± 1.316^ab^
10 mg/kg PM2.5 group	9.54 ± 0.47^abc^	64.45 ± 11.98^abcd^	24.80 ± 3.04^ab^	10.556 ± 1.699^abc^
*F*	24.556	6.708	6.774	21.053
*p*	<0.001	<0.001	<0.001	<0.001

Note: compared to the control group, ^a^*p* < 0.05; compared to the 0.75 mg/kg PM2.5 group, ^b^*p* < 0.05; compared to the 2.5 mg/kg PM2.5 group, ^c^*p* < 0.05; compared to the 5 mg/kg PM2.5 group, ^d^*p* < 0.05.

**Table:** (b) Intraperitoneal glucose tolerance test in rats (IPGTT) ($\overset{®}{x}\pm s$)

	Blood glucose (mmol/L)						AUC_glu_
	0′	5′	10′	30′	60′	120′	
A	5.2 ± 0.3	6.3 ± 0.4	6.9 ± 0.5	8.6 ± 0.9	7.8 ± 1.0	5.5 ± 0.3	11.1 ± 0.9
B	6.0 ± 0.5	8.4 ± 0.7	9.8 ± 1.1	11.4 ± 0.9	9.8 ± 1.7	7.4 ± 1.6	14.5 ± 1.2^a^
C	7.5 ± 1.5^ab^	11.2 ± 1.0^ab^	12.2 ± 0.7^a^	12.7 ± 1.4^a^	11.1 ± 2.6^ab^	7.1 ± 0.7^a^	16.4 ± 0.9^ab^
D	8.5 ± 0.7^abc^	13.5 ± 3.1^ab^	17.1 ± 4.4^abc^	17.5 ± 1.8^abc^	9.7 ± 1.7^ab^	7.8 ± 2.2^a^	19.1 ± 1.4^abc^
E	9.5 ± 0.5^abc^	12.8 ± 2.9^ab^	15.4 ± 3.4^ab^	19.2 ± 2.0^abc^	15.0 ± 2.9^abc^	10.3 ± 0.7^abcd^	22.8 ± 1.8^abcd^
*F*	24.56	11.78	12.95	43.22	8.21	9.16	60.61
*p*	<0.001	<0.001	<0.001	<0.001	<0.001	<0.001	<0.001

Note: A: control group; B: 0.75 mg/kg PM2.5 group; C: 2.5 mg/kg PM2.5 group; D: 5 mg/kg PM2.5 group; E: 10 mg/kg PM2.5 group; AUC_glu_: area under the curve; *F*: *F* value; *p*: *p* value. Compared to the control group, ^a^*p* < 0.05; compared to the 0.75 mg/kg PM2.5 group, ^b^*p* < 0.05; compared to the 2.5 mg/kg PM2.5 group, ^c^*p* < 0.05; compared to the 5 mg/kg PM2.5 group, ^d^*p* < 0.05.

**Table:** (a) Serum concentrations of ALT, AST, TBIL, IL-6, and TNF-*α* in rats ($\overset{®}{x}\pm s$)

	ALT (U/L)	AST (U/L)	TBIL (*μ*mol/L)	IL-6 (pg/mL)	TNF-*α* (pg/mL)
Control group	126.1 ± 13.9	218.7 ± 39.1	8.77 ± 1.05	104.1 ± 15.5	177.7 ± 31.7
0.75 mg/kg PM2.5 group	136.9 ± 18.4	237.2 ± 23.4	9.02 ± 0.92	110.8 ± 30.1	211.5 ± 24.5
2.5 mg/kg PM2.5 group	182.2 ± 17.3^ab^	282.7 ± 23.3^a^	10.14 ± 1.18	163.4 ± 29.5^ab^	232.1 ± 38.4^a^
5 mg/kg PM2.5 group	250.0 ± 18.1^abc^	374.4 ± 38.6^abc^	13.22 ± 1.70^abc^	202.5 ± 44.9^ab^	290.0 ± 27.4^abc^
10 mg/kg PM2.5 group	281.8 ± 29.4^abcd^	422.3 ± 43.6^abcd^	17.36 ± 1.49^abcd^	222.6 ± 40.8^abc^	347.3 ± 61.5^abcd^
*F*	58.359	32.314	38.886	12.346	14.832
*p*	<0.001	<0.001	<0.05	<0.05	<0.001

Note: compared to the control group, ^a^*p* < 0.05; compared to the 0.75 mg/kg PM2.5 group, ^b^*p* < 0.05; compared to the 2.5 mg/kg PM2.5 group, ^c^*p* < 0.05; compared to the 5 mg/kg PM2.5 group, ^d^*p* < 0.05.

**Table:** (b) Changes in liver IL-6 and TNF-*α* gene expression in rats ($\overset{®}{x}\pm s$)

	IL-6	TNF-*α*
Control group	0.982 ± 0.231	1.009 ± 0.158
0.75 mg/kg PM2.5 group	1.009 ± 0.202	0.986 ± 0.166
2.5 mg/kg PM2.5 group	1.185 ± 0.243	1.333 ± 0.208^ab^
5 mg/kg PM2.5 group	1.641 ± 0.207^abc^	1.714 ± 0.259^abc^
10 mg/kg PM2.5 group	2.048 ± 0.172^abcd^	2.128 ± 0.199^abcd^
*F*	23.527	29.364
*p*	<0.001	<0.001

Note: compared to the control group, ^a^*p* < 0.05; compared to the 0.75 mg/kg PM2.5 group, ^b^*p* < 0.05; compared to the 2.5 mg/kg PM2.5 group, ^c^*p* < 0.05; compared to the 5 mg/kg PM2.5 group, ^d^*p* < 0.05.

**Table 4:** Changes in liver expression of INSR, IRS-1, PI3K, AKT, and GLUT2 in rats ($\overset{®}{x}\pm s$).

	INSR	IRS-1	PI3K	AKT	GLUT2
Control group	1.053 ± 0.135	1.193 ± 0.201	1.254 ± 0.186	1.195 ± 0.231	1.185 ± 0.247
0.75 mg/kg PM2.5 group	1.014 ± 0.146	1.000 ± 0.134	1.117 ± 0.196	1.021 ± 0.198	1.009 ± 0.159
2.5 mg/kg PM2.5 group	0.997 ± 0.217	1.043 ± 0.185	0.967 ± 0.333	1.051 ± 0.226	0.938 ± 0.243^a^
5 mg/kg PM2.5 group	0.691 ± 0.124^abc^	0.798 ± 0.111^abc^	0.855 ± 0.227^a^	0.723 ± 0.206^abc^	0.763 ± 0.050^ab^
10 mg/kg PM2.5 group	0.605 ± 0.172^abc^	0.651 ± 0.171^abc^	0.685 ± 0.150^ab^	0.645 ± 0.087^abc^	0.599 ± 0.143^abc^
*F*	8.210	8.434	4.753	7.026	7.571
*p*	<0.001	<0.001	<0.001	<0.001	<0.001

Note: compared to the control group, ^a^*p* < 0.05; compared to the 0.75 mg/kg PM2.5 group, ^b^*p* < 0.05; compared to the 2.5 mg/kg PM2.5 group, ^c^*p* < 0.05; compared to the 5 mg/kg PM2.5 group, ^d^*p* < 0.05.

**Table 5:** Changes in the p-IRS-1/IRS-1 and p-AKT/AKT ratios in the rat liver ($\overset{®}{x}\pm s$).

	p-IRS-1/IRS-1	p-AKT/AKT
Control group	0.215 ± 0.041	0.661 ± 0.041
0.75 mg/kg PM2.5 group	0.344 ± 0.038^a^	0.653 ± 0.056
2.5 mg/kg PM2.5 group	0.536 ± 0.036^ab^	0.544 ± 0.098^ab^
5 mg/kg PM2.5 group	0.724 ± 0.076^abc^	0.370 ± 0.083^abc^
10 mg/kg PM2.5 group	0.800 ± 0.018^abcd^	0.261 ± 0.046^abcd^
*F*	144.861	33.347
*p*	<0.001	<0.001

Note: compared to the control group, ^a^*p* < 0.05; compared to the 0.75 mg/kg PM2.5 group, ^b^*p* < 0.05; compared to the 2.5 mg/kg PM2.5 group, ^c^*p* < 0.05; compared to the 5 mg/kg PM2.5 group, ^d^*p* < 0.05.

## Data Availability

The data used to support the findings of this study are available from the corresponding author upon request.
